# Identification of coding sequence and its use for functional and structural characterization of catalase from *Phyllanthus emblica*

**DOI:** 10.6026/97320630014008

**Published:** 2018-01-31

**Authors:** Swati Sharma, Vinita Hooda

**Affiliations:** 1 Department of Botany, Faculty of Life Sciences, Maharshi Dayanand University, Rohtak-124001, India;

**Keywords:** Catalase, Antioxidant enzyme, Phyllanthus emblica, GenBank, InterProScan, Multiple sequence alignment, Phylogenetic analysis

## Abstract

Catalase is an essential antioxidant enzyme that is well characterized from microbial and animal sources. The structure of plant catalase is unknown. Therefore, it is of interest to understand the functional and structural characteristics of catalase from an Indian gooseberry, Phyllanthus emblica (or Emblica officinalis). Hence, catalase from P. emblica was cloned in pUC18 plasmid, sequenced and submitted to GenBank with the accession numbers "MF979112" and "ATO98311.1". InterProScan showed that the coding sequence is monofunctional and haem-dependent catalase-like superfamily. Multiple sequence alignment (MSA) followed by phylogenetic analysis showed that the P. emblica catalase groups with soybean catalase. We further report the characteristics of structural model of the enzyme for functional characterization.

## Background

Free radicals including reactive oxygen species (ROS) are regularly generated as byproducts of various metabolic reactions in a cell. Excessive release of ROS damage proteins, lipids, and DNA, which causes oxidative stress that eventually, leads to functional loss of a cell and apoptosis [1]. To counter the toxic effects of ROS, the eukaryotic cell produces various antioxidant enzymes including peroxidase, superoxide dismutase, polyphenol oxidase, catalase etc. Out of these enzymes, catalase is considered to be a highly active key antioxidant enzyme [2] that reduces oxidative stress by catalyzing the conversion of hydrogen peroxide to water and oxygen [3]. Moreover, this enzyme shows a very high apparent Km in the range of 0.025 - 1722 mM and hence is not easily saturated with its substrate [4].

Catalases have been purified and structurally characterized from various microbial [5, 6, 7, 8, 9] and animal sources [10]. However, limited understanding of catalases function from rice [11] and wheat [12] is known using structural and functional data. Nonethless, structural information on plant catalases is limited and needs to be explored further [13]. Phyllanthus emblica (P. emblica; common name: Gooseberry) is known to be an excellent source of antioxidants and hence was presumed to be rich in catalases too. Therefore, it is of interest to characterize catalase from P. emblica using structural models.

## Methodology

### Materials and machines used

Cloning vector (pUC18) and E.coli strain DH5α, DNA ladder, protein molecular weight marker and restriction endonucleases (EcoR1 and HindIII) were obtained from Genei laboratories Pvt. Ltd., India. RNA isolation and cDNA synthesis were accomplished using RNAsolTM and the first strand cDNA synthesis kits respectively from Chromous Biotech, India. All other chemicals of analytical reagent grade from HiMedia, India were used.

Polymerase chain reactions (PCR) were performed with peqSTAR96 universal gradient thermal cycler, Avantor, U.S.A. Other instruments used were BioRad Mini-Protean Tetra System for gel electrophoresis, U.S.A and BioRad Gel Doc EZ imager, U.S.A for capturing the images of gel. Sequencing was done with ABI 3500 Genetic analyzer at Chromous Biotech, India. The computational work was done on Intel(R) core, 2.20 GHz, 32-bit operating system.

### Cloning of catalase gene

RNA was isolated from the freshly plucked young leaves of a healthy P.emblica plant and used to synthesize the first strand of cDNA. This cDNA was used to amplify the catalase coding sequence (CDS) or the catalase gene with PCR, using catalase specific primers (Figure 7) at initial denaturation of 5 min at 94°Cfollowed by 35 cycles of denaturation, annealing and elongation at 94°C, 55°C and 72°C respectively. The purified-gel fragment was ligated to pUC18 cloning vector at EcoRI and HindIII cloning sites after confirmation of its sequence and clone in E.coli DH5α. Probable clones were screened by colony PCR. The cloned catalase CDS was further digested with EcoRI/HindIII restriction enzymes. The size of catalase insert released from the pUC18 vector was analyzed on agarose gel. Further, it was sequenced to confirm its identity.

### Computational analysis of P. emblica catalase gene

The coding sequence obtained from P. emblica was confirmed using BLAST and translated into protein sequence using the ExPASy translate tool [14].

### Protein Annotation

Protein annotation was done by InterProScan protein domain identifier [15] by scanning the databases such asprosite profiles, panther, SMART (Simple Modular Architecture Research Tool), Pfam and Gene3D for conserved domain identification. Multiple sequence alignment (MSA) was done using Clustal Omega (1.2.4) multiple alignment tool and a phylogenetic tree of isozymesof plant catalases available at UniProtKB database was constructed using Molecular Evolutionary Genetics Analysis tool MEGA6.06.

### Structure prediction and refinement

The secondary structure features of P. emblica catalase was analyzed by Self-Optimized Prediction method with Alignment (SOPMA) [16] and its 3D structure was predicted based on template-based modeling by I-tasser (Iterative Threading ASSEmbly Refinement) server [17]. The threading templates chosen by the I-Tasser server from the PDB database on the basis of normalized Z-score of >1.0 were, 1QWL (Helicobacter pyloricatalase), 2ISA (Vibrio salmonicidia catalase), 4QOL (Bacillus pumilus catalase), 2J2M (Exiguobacterium oxidotolerans catalase), 4AUM (Scytalidium thermophilum catalase) and1DGF (human erythrocyte catalase). Then, by reassembling fragments excised from threading templates, ten different 3D structural models were constructed by I-tasser.

The energy of 3D models was minimized using GalaxyRefine web server [18, 19]. The successful refinement of the structure by this method is driven by side chain repacking and relaxing the overall structure by molecular dynamics simulation, which provides more precise structures for the structural and functional study of the protein.

## Results and discussion

### cDNA synthesis and sequencing

**Figure 1:**
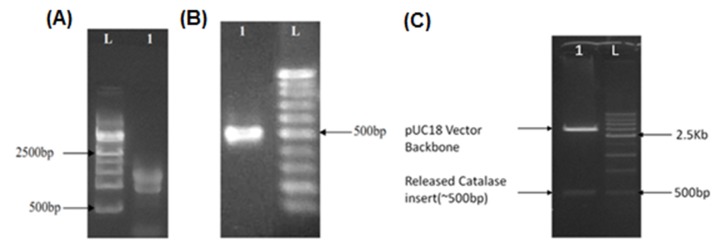
(A) Total RNA isolated from leaves of P.emblica: Lane description: L- 500 bp DNA ladder; 1- Total RNA from P.emblica leaf. (B) Band in the lane1 showing the amplified catalase cDNA after loading onto 2% agarose gel; L- 100 bp DNA ladder. (C) Digestion confirmation of pUC18+catalase clone (digested with EcoRI/HindIII). 1C). The released catalase insert is seen in lane 1 against the 500 bp long DNA fragment in the ladder lane (L).

The agarose gel image presented in Figure 1A confirmed the isolation of RNA from P. emblica leaves (Figure 1A). The cDNA synthesized from the purified RNA was analyzed on agarose gel and found to be approximately 500 bp long (Figure 1B). The purified-gel fragment was then sequence confirmed and then cloned into the initial cloning vector, pUC18. Colony PCR screening and further digestion of the plasmids with restriction enzymes confirmed the presence of catalase insert (Figure 1C). The released catalase insert was found to be 510 bp long when sequenced by Sanger's dideoxy sequencing. A high similarity of the submitted CDS with other catalases (87% similarity with CDS of Populus trichocarpa catalase; sequence ID: XM_002306940.2) via nucleotide BLAST at NCBI established its identity as catalase. The P. emblica catalase CDS has been submitted to GenBank (NCBI) with accession No. MF979112. The 170 amino acid long sequence deduced from this partial cDNA sequence is also available at NCBI with the protein_id"ATO98311.1"

### Characterization of translated catalase CDS

BLASTP of translated CDS revealed a pretty high 96% identity with other homologous sequences (Table 1). InterProScan matched the P. emblica translated CDS against the signatures from various other databases such as prosite profiles, panther, SMART, Pfam and GENE3D and the results confirmed that the derived amino acid sequence from P. emblica belonged to monofunctional, haem-dependent catalase-like superfamily.

### Multiple sequence alignment

**Figure 2:**
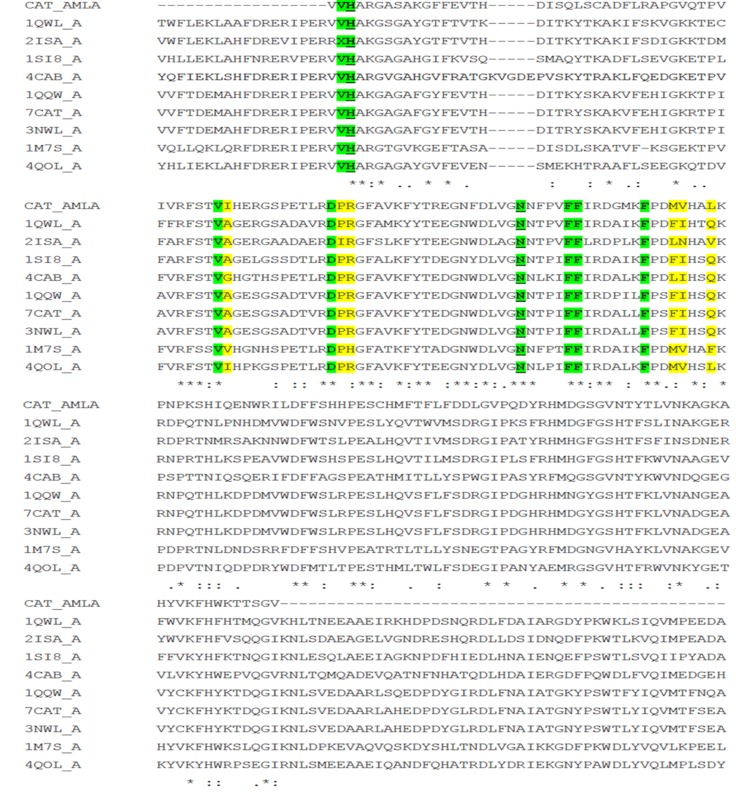
Multiple sequence alignment (MSA) of catalase sequence derived from P. emblica (CAT_Amla) with 9 homologous sequences of Protein Data Bank (PDB) using CLUSTAL Omega (1.2.4). The residues involved in the hydrogen peroxide binding are highlighted in green color and the substituted residues are highlighted in yellow color. Conserved residues playing a key role in hydrogen peroxide binding as identified by Prosite-ProRule annotation are marked as bold and underlined with green color highlight.

Clustal Omega (1.2.4) was used for multiple sequence alignment (MSA) and active site identification. An alignment of the translated CDS of P. emblica catalase with catalases from other sources is shown in Figure 2. Conserved residues of the catalase sequence involved in the H2O2 binding (V 2, H 3, V 44, D 56, N 76, F 81, F 82, F 89) were identified after carefully studying the alignment. The results were found to be consistent with the experimentally determined crystallographic structures of human erythrocyte catalase (1QQW) [6] and Deinococcus radiodurans catalase (4CAB) [9]. However, few substitutions such as of isoleucine (I) by alanine (A), of methionine (M) by phenylalanine (F), of valine (V) by isoleucine (I) and of glutamine (Q) by leucine (L) were also observed in the translated CDS of catalase.

**Figure 3:**
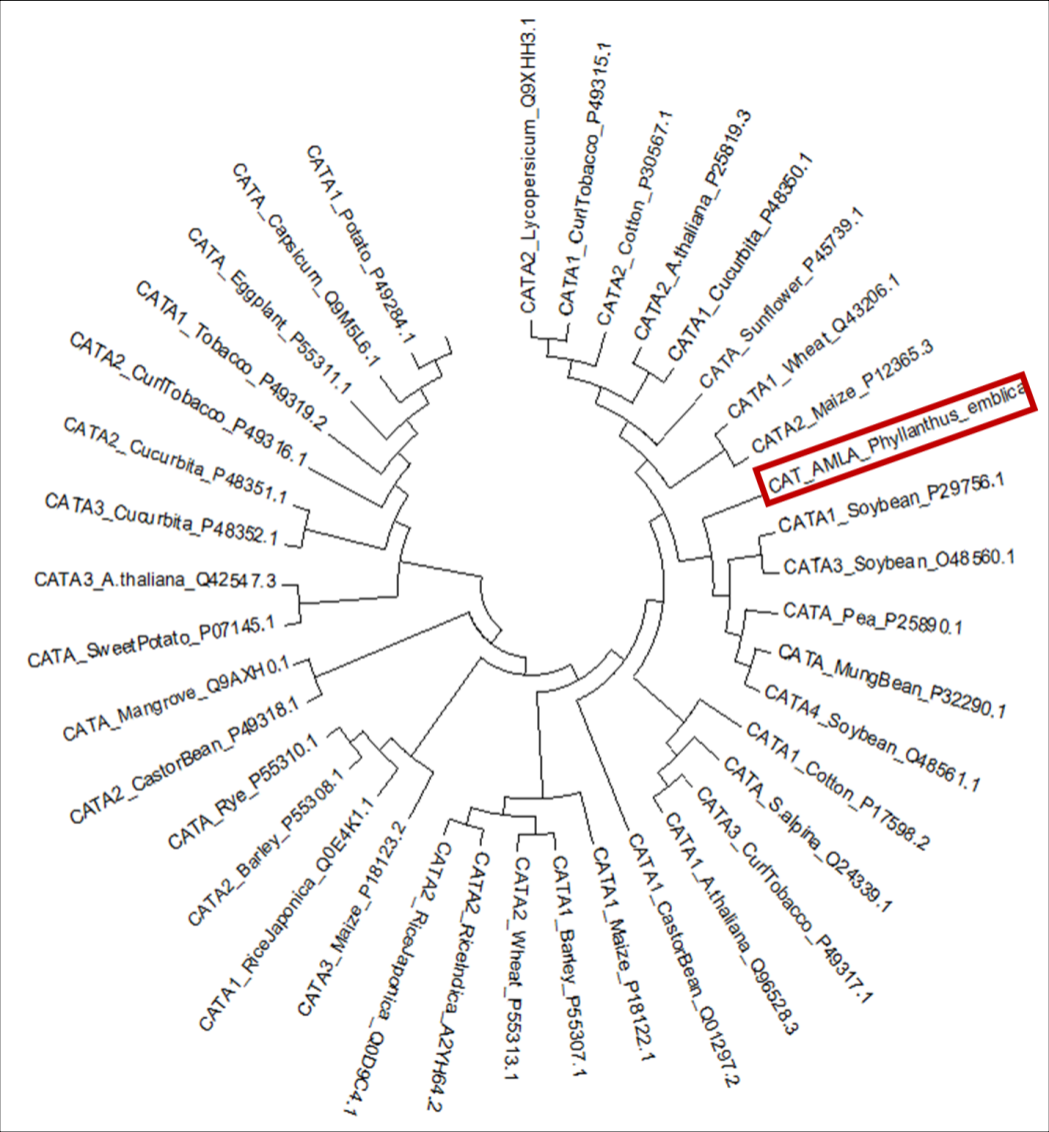
A phylogenetic tree of plant catalase isozyme sequences available at UniProtKB database, constructed using (Molecular Evolutionary Genetics Analysis) MEGA 6.06. clustered P. emblica catalase CDS with catalases from other plant sources viz. soybean, pea, and mung bean.

A phylogenetic tree was also constructed using MEGA 6.06 to know the evolutionary relatedness of the P. emblica catalase CDS with all other isozymes of plant catalases available at UniProtKB database. Though, the translated catalase CDS clustered with a branch of several plants, including soybean, pea, and mung bean but was found to be phylogenetically closest to the catalase (CATA1) from soybean (Figure 3).

### Structural characterization of the translated catalase CDS

The secondary structure features as predicted by Self-Optimized Prediction method with Alignment (SOPMA) shows that random coils (35.88%) dominated among secondary structure elements followed by extended strands (28.82%), beta-turn (18.82%) and the alpha helix (16.47%). The predominance of coils points to the fact that catalase from P. emblica might not be a very stable enzyme [20].

### 3D model building, refinement, and evaluation

**Figure 4:**
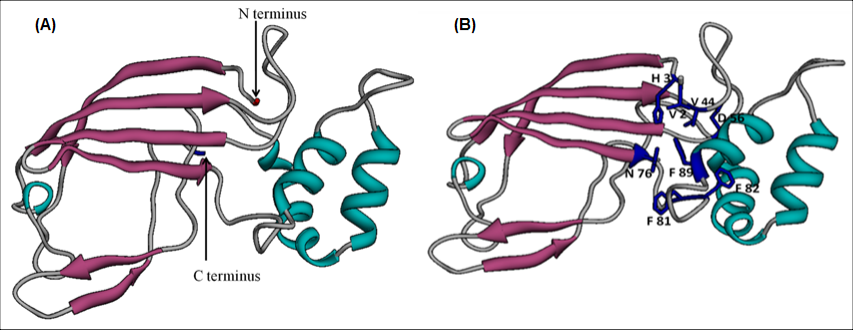
(A) Ribbon model of P. emblica catalase CDS as visualized by Chimera1.5.1. (B) 3D model of P. emblica catalase CDS showing the labeled residues for H2O2binding (V 2, H 3, V 44, D 56, N 76, F 81, F 82, F 89) in blue color.

**Figure 5:**
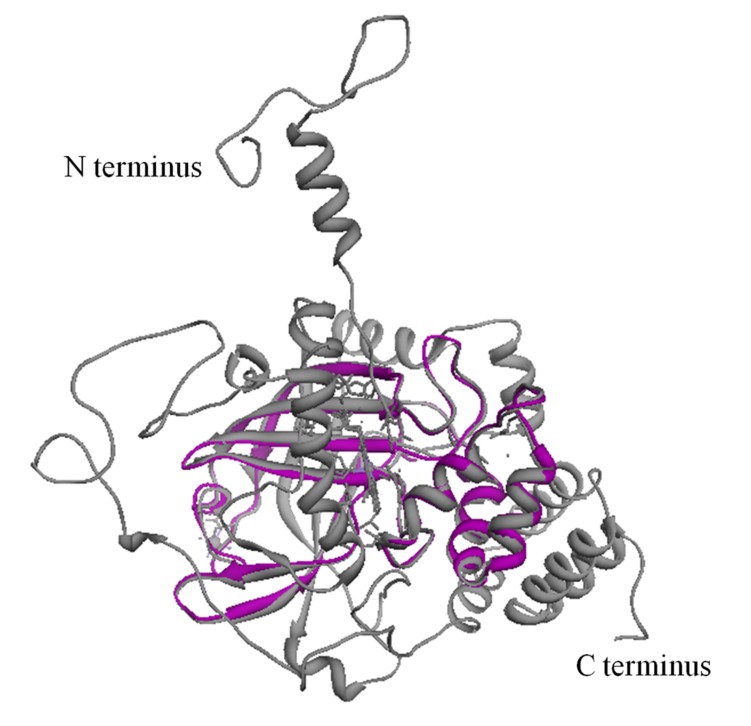
Structural superimposition of the partial P. emblica catalase CDS (magenta color) with the chain A (light grey color) of template 4QOL (Bacillus pumilus catalase) as visualized with Chimera1.5.1 having RMSD value of 0.589.

The 3D model of the P. emblica partial catalase sequence was built by I-tasser server is depicted in Figure 4A. The quality of 3D model was assessed on the basis of the confidence score (C-score: 1.60), which is well within the range (-5 to 2). Minimizing the energy using GalaxyWeb server refined the model build. The validated model using various programs such as Ramachandran plot, ERRAT, Verify-3D, ProSaWeb Z-score and energy plot confirmed the reliability of the model. All the parameters for validation were within the range showing the compatibility of the model with its sequence and depicting the excellent quality model. Structural alignment of the predicted model with the template in Figure 5 has very low RMSD (0.589) showing reliability of the experimental structure for the functional annotation of the predicted model.

### Surface analysis of the model

The surface analysis of the model was done using solvent accessibility score and electrostatic potential map. Solvent accessibility (SA) prediction score, ranging from 0 (buried residue) to 9 (highly exposed residue) was calculated using Itasser server [17]. The SA score for H2O2 binding residues was found to be: V2 "3"; H3 "0"; V44 "1"; I45 "3"; D56 "2"; P57 "2"; R58 "3"; N76 "3"; F81 "0"; F82 "0"; F89 "1"; M92 "2"; V93 "1" and L96 "2". Low values clearly showed that the active site amino acid residues lied within the crevices or the cleft, hence reinforcing that they might be the part of catalytic site.

**Figure 6:**
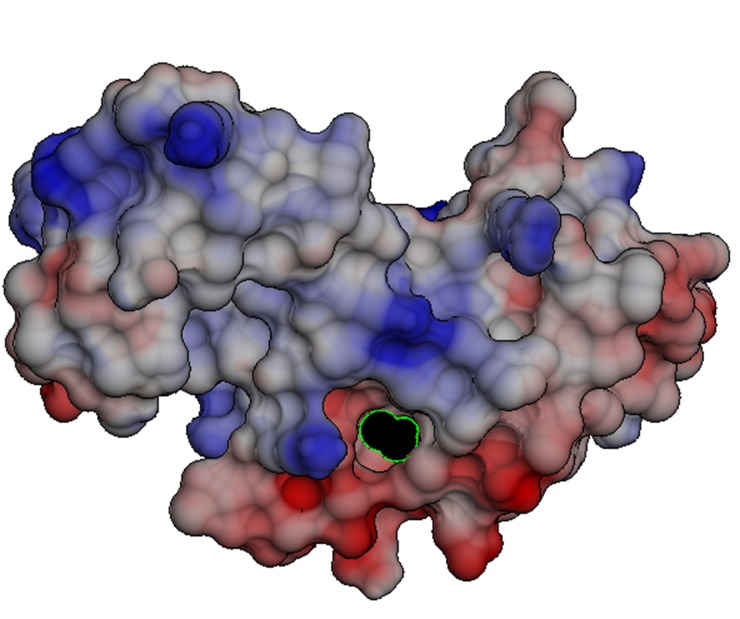
Electrostatic surface analysis of the model using Chimera 1.4.1: a color spectrum with red as the lowest electrostatic potential energy value and blue as the highest. Ligand (H2O2) is shown as black sphere with green boundary, binding at the high electron density active site groove shown in red color.

**Figure 7:**
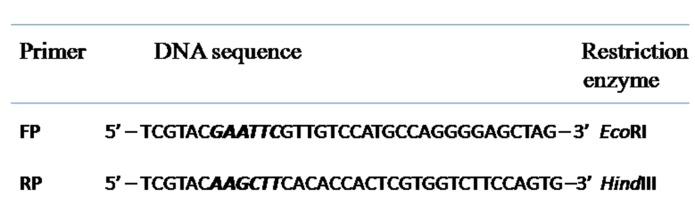
PCR primers used for P.emblica catalase CDS amplification: FP: Forward Primer; RP: Reverse Primer. EcoR1 restriction sites in the FP and HindIII restriction sites in the RP are marked in italics

To visualize the charge distributions of molecules, electrostatic potential maps are very useful. To make the electrostatic potential energy data easy to interpret, a color spectrum, with red as the lowest electrostatic potential energy value and blue as the highest, is employed by chimera 1.5.1 to convey the varying intensities of the electrostatic potential energy values. Here, the red color binding cleft (Figure 6) shows the lowest electrostatic potential corresponding to the area of greatest electron concentration. Hence, the groove constitutes a perfect active site, which attracts the ligand, H2O2 (displayed as black sphere with green boundary in the Figure 6) towards itself. Electrostatic potential maps were generated using chimera 1.5.1 to know the charge distribution of molecules. The Figure 6 represents red to blue regions in the order of decreasing electron densities. As is evident from the Figure 6, the ligand (H2O2) sits more towards the red area lined by the predicted active site residues. Since, the catalytic site is actively involved in charge transfer reactions required for formation and degradation of bonds, so it is expected to have high electron density [23].

## Conclusion

It is of interest to understand the functional and structural characteristics of Phyllanthus emblica. We deposited the catalases coding sequence (CDS) at GenBank. InterProScan shows the sequence is of a mono functional haem-containing catalase. Conserved key residues involved in substrate catalysis were shown using multiple sequence alignment grouped with the catalase from P. emblica after phylogentic analysis. A structural model of the plant catalase and its surface analysis was reported for further functional characterization.

## Figures and Tables

**Table 1:** Protein Blast of target sequence, P.emblica catalase with nonredundant database; top 10 protein sequences on the basis of E-value and % identity are displayed

Accession No.	Source of catalase	E value	% Identity
AHG98056.1	*Plectranthus barbatus*	1.00E-117	96
AAX88799.1	*Euphorbia characias*	4.00E-115	95
AIA61608.1	*Gynostemma pentaphyllum*	4.00E-115	95
NP_00314123.1	*Gossypium hirsutum*	4.00E-115	96
NP_001291326.1	*Sesamum indicum*	8.00E-115	95
AKN08992.1	*Luffa aegyptiaca*	1.00E-114	95
NP_001310800.1	*Ziziphus jujube*	1.00E-114	95
XP_021902483.1	*Carica papaya*	2.00E-114	95
NP_001289779.1	*Nelumbo nucifera*	2.00E-114	95
XP_022132848.1	*Momordica charantia*	3.00E-114	94
